# Predictive Value of Systemic Immune-inflammation Index in Determining Mortality in COVID-19 Patients

**DOI:** 10.2478/jccm-2022-0013

**Published:** 2022-08-12

**Authors:** Tahsin Karaaslan, Esra Karaaslan

**Affiliations:** 1 Medeniyet University Göztepe Training and Research Hospital, Istanbul, Turkey; 2 Istanbul Medipol University, Istanbul, Turkey

**Keywords:** COVID-19, mortality, systemic immune-inflammation index, neutrophil/lymphocyte ratio, inflammatory markers

## Abstract

**Aim:**

The aim of this study was to evaluate whether systemic immune-inflammation index (SII) could predict mortality in patients with novel coronavirus 2019 (COVID-19) disease.

**Methods:**

This two-center, retrospective study included a total of 191 patients with confirmed diagnosis of COVID-19 via nucleic acid test (NAT). The SII was calculated based on the complete blood parameters (neutrophil × platelet/lymphocyte) during hospitalization. The relationship between the SII and other inflammatory markers and mortality was investigated.

**Results:**

The mortality rate was 18.3%. The mean age was 54.32±17.95 years. The most common symptoms were fever (70.7%) and dry cough (61.3%), while 8 patients (4.2%) were asymptomatic. The most common comorbidities were hypertension (37.7%), diabetes (23.0%), chronic renal failure (14.7%), and heart failure (7.9%) which all significantly increased the mortality rate (p<0.001). There was a highly positive correlation between the SII and polymorphonuclear leukocyte (PNL), neutrophil-to-lymphocyte ratio (NLR), and platelet-to-lymphocyte ratio (PLR) (r=0.754, p<0.001; r=0.812, p<0.001; r=0.841, p<0.001, respectively), while a moderate, positive correlation was found between the SII and C-reactive protein (CRP) (r=0.439, p<0.001). There was a significant correlation between the SII and mortality (U=1,357, p<0.001). The cut-off value of SII was 618.8 (area under the curve=0.751, p<0.001) with 80.0% sensitivity and 61.5% specificity. A cut-off value of >618.8 was associated with a 4.68-fold higher mortality.

**Conclusion:**

Similar to NLR and PLR, the SII is a proinflammatory marker of systemic inflammation and can be effectively used in independent predicting COVID-19 mortality.

## Introduction

The first cases of novel coronavirus 2019 (COVID-19) caused by severe acute respiratory syndrome-coronavirus-2 (SARS-CoV-2) was identified in Wuhan, Hubei province of China in December 2019. On January 9^th^, 2020, the virus was identified as a coronavirus belonging to the family *Coronaviridae* [1]. The COVID-19 rapidly evolved into a global outbreak and the World Health Organization (WHO) declared the outbreak as a pandemic on March 11^th^, 2020 [2].

The disease has a wide range of clinical presentations from asymptomatic and mild symptoms to severe respiratory failure and mortality. Fever, dry cough, dyspnea, fatigue, and myalgia are the typical symptoms [3]. The diagnosis is made based on clinical, laboratory, and imaging study findings. However, the diagnosis is confirmed by nucleic acid test (NAT) which detects the particular nucleic and sequence of the virus. In a variety of samples, nucleic acid material has been confirmed by polymerase chain reaction (PCR). In daily practice, PCR is performed using the nasopharyngeal swabs; however, SARS-CoV-2 can be detected in bronchoalveolar lavage fluid, urine, or anal swabs [4].

Prompt diagnosis is of utmost importance for early treatment, early isolation, and early admission to intensive care unit (ICU), when necessary. Identification of patients who are at risk for critical illness and mortality is also vital to tailor optimal treatment and follow-up strategies. To date, several predictors have been proposed to be useful in assessing the risk of critical illness and mortality including absolute lymphocyte count, neutrophil-to-lymphocyte ratio (NLR), platelet-to-lymphocyte ratio (PLR), red blood cell distribution width (RDW), C-reactive protein (CRP), ferritin, troponin, D-dimer, and creatinine [5, 6, 7].

The systemic immune-inflammation index (SII) is calculated based on the neutrophil × platelet/lymphocyte count. The SII has been widely used to predict the prognosis of certain types of malignancy [8, 9, 10]. However, its clinical significance in infections has not been fully elucidated yet. In the present study, we aimed to evaluate whether SII could predict mortality in patients with COVID-19 disease.

## Materials and methods

### Study design and study population

This retrospective study included pacients admitted to the Department of Internal Medicine and Chest Diseases of two centers between May 1st, 2020 and February 28th, 2021. Patients aged above 18 years whose NAT reverse transcriptase-PCR/NAT result was positive using nasopharyngeal swabs were included in the study. Patients having hematological or solid organ malignancy, receiving immunosuppressive treatment for any reason, PCR/NAT-negative cases, and missing radiological, biochemical or clinical findings were excluded. A written informed consent was obtained from each patient for all diagnostic and therapeutic procedures. The study protocol was approved by the institutional Ethics Committee (Date: 18.03.2021/No: 2021/331). The study was conducted in accordance with the principles of the Declaration of Helsinki.

### Data collection

Data of the patients were retrieved from the electronic databases of both centers. Data including age, sex, comorbidities such as hypertension, diabetes, heart failure, or renal failure, symptoms at the time of admission, biochemical parameters, thoracic computed tomography (CT) findings, and complete blood counts were recorded. All data were obtained at the time of hospital admission. The patients were divided into two groups as survivors (n=156) and non-survivors (n=35).

At the time of admission, complete blood count analysis including hemoglobin level, leukocyte count, platelet count (PLT), absolute neutrophil count, eosinophil count, and RDW was performed for each patient. The NLR, PLR, and SII (neutrophil × platelet/lymphocyte) were calculated. Estimated glomerular filtration rate (eGFR) was calculated using the Chronic Kidney Disease Epidemiology Collaboration (CKD-EPI) formula. All CT images were acquired at the end of inhalation using a 16-slice CT scanner (Somatom™ Scope Power; Siemens Healthineers, Forchheim, Germany).

### Statistical analysis

Statistical analysis was performed using the SPSS version 26.0 software (IBM Corp., Armonk, NY, USA). Descriptive data were expressed in mean ± standard deviation (SD), median (min-max) or number and frequency, where applicable. The Student *t*-test was used to compare normally distributed variables between the groups, while the Mann-Whitney U test was performed to compare non-normally distributed variables between the groups. Categorical variables were analyzed using the chi-square test. Pearson correlation coefficient was used to analyze the correlation between the SII and COVID-19 mortality. The receiver operating characteristics (ROC) curve was used to determine the cut-off value and sensitivity and specificity of SII. The independent effect of SII on mortality was examined by multivariate logistic regression analysis. A *p* value of <0.05 was considered statistically significant.

## Results

A total of 191 patients including 126 (66.0%) from the first center and 65 (34.0%) from the second center were included in the study. Of these patients, 35 (18.3%) died. Baseline demographic characteristics and clinical and radiological findings of the patients are summarized in Table 1.

**Table 1:** Demographic characteristics and clinical and radiological findings of the patients.

		N	Mean			Value	p
Age (year)	Total	191	54,32±17,95	min= 20,	max: 95		
	Survivor	156	50,10±15,76	min= 20,	max: 93	t= -7,88	<0,001
	Non-survivor	35	73,11±14,94	min= 23,	max: 95		
		N	%	Mortality			
Sex	Female	97	50.8%	n=18	18.6%		
	Male	94	49.2%	n=17	18.1%	χ2=0,007	0,933

Center	First center	126	66.0%	n=32	25.4%		
	Second center	65	34.0%	n=3	4.6%	χ2=12,37	<0,001

Symptoms	Yes	183	95.8%	n=35	19.1%		0,355*
	No	8	4.2%	n=0	0.0%		

Fever	Yes	135	70.7%	survivor	n=111; 71.2%		
	No	56	29.3%	non-survivor	n=24; 68.6%	χ2=0,092	0,762

Cough	Yes	117	61.3%	survivor	n=95; 60.9%		
	No	74	38.7%	non-survivor	n=22; 62.9%	χ2=0,046	0,830

Fatigue	Yes	108	56.5%	survivor	n=87; 55.8%		
	No	83	43.5%	non-survivor	n=21; 60.0%	χ2=0,208	0,648

Dyspnea	Yes	64	33.5%	survivor	n= 40; 25.6%	χ2=23,65	<0,001
	No	127	66.5%	non-survivor	n= 24; 68.6%		

Myalgia	Yes	53	27.7%	survivor	n= 48; 30.8%		
	No	138	72.3%	non-survivor	n=5; 14.3%	χ2=3,87	0,049

Diarrhea	Yes	31	16.2%	survivor	n=25; 16.0%		
	No	160	83.8%	non-survivor	n=6; 17.1%	χ2=0,026	0,871

Loss of taste	Yes	16	8.4%	survivor	n=13; 8.3%	χ2=0,02	0,963
and smell	No	175	91.6%	non-survivor	n=3; 8.6%		

Thoracic CT	Positive	168	88.0%	Mortality	20.3%		0,009*
scan	Negative	23	12.0%	Mortality	0.0%		
	Ground-opacity glass	164	85.9%	Mortality n=33	20.1%	χ2=2,50	0,114
	Consolidation	68	35.6%	Mortality n=20	29.4%	χ2=8,67	0,003

χ2=Pearson Chi-Square, t=t-test for Equality of Means, * Fisher’s Exact Test

Of the patients, 97 (50.8%) were females and the mean age was 54.32±17.95 (range, 20 to 95) years. No mortality was observed in the patients having a normal CT scan (n=23, 12.0%), while 20.8% (n=35) of the patients having abnormal CT findings. In the subgroup analysis, there was no significant correlation of ground-glass appearance and pleural effusion with mortality (p>0.05). However, 29.4% (n=20) of the patients with consolidation on CT died, indicating statistical significance (χ2=8.68, p=0.003) (Table 1).

The most common symptoms were fever (70.7%), dry cough (61.3%), fatigue (56.5%), and dyspnea (33.5%). No significant correlation was observed between the symptoms and mortality, while there was a significant correlation between dyspnea and mortality (χ2=23.65, p<0.001). On the contrary, the mortality rate was significantly lower among the patients with myalgia (χ2=3.87, p=0.049). No mortality was observed in asymptomatic patients (4.2%) (Table 1).

A total of 16.2% (n=31) of the patients who had normal renal functions at the time of admission developed acute kidney injury (AKI), and nine of them required hemodialysis. Totally, 66.7% (n=6) of these patients died. The most common comorbidities were hypertension (37.7%), diabetes (23.0%), chronic renal failure (14.7%), and heart failure (7.9%) which all significantly increased the mortality rate (p<0.001) (Table 2).

**Table 2:** Comorbidities of patients

	N	%			Value	p
AKI	31	16.2 %	survivor	n=16; 10.3%	χ2=22,35	<0,001
			non-survivor	n=15; 42.9%		

Renal Tx	6	3.1 %	survivor	n=5; 3.2%		1,000
			non-survivor	n=1; 2.9%		(Fisher’s)

CKD	28	14.7 %	survivor	n=10; 6.4%	χ2=46,31	<0,001
			non-survivor	n=18; 51.4%		

Hypertension	72	37.7 %	survivor	n= 42; 26.9%	χ2=42,07	<0,001
			non-survivor	n= 30; 85.7%		

Diabetes	44	23.0 %	survivor	n= 26; 16.7%	χ2=19,48	<0,001
			non-survivor	n= 18; 51.4%		

Heart failure	15	7.9 %	survivor	n= 7; 4,5%	χ2=13,33	<0,001
			non-survivor	n= 8; 22,9%		
	median	Min.-max.	median		U	p
Creatinine	0,88	0,44-15,36	survivor	0,825	1230,5	<0,001
(mg/dL)			non-survivor	1,52		

eGFR (CKD-Epi)	88,13	3,02-133,44	survivor	94,19	808,5	<0,001
(ml/dk)			non-survivor	41,74		

LDH	343,0	155-25000	survivor	284,0	779,5	<0,001
(U/L)			non-survivor	408,0		

CRP	9,81	0,36-35,25	survivor	5,80	856,5	<0,001
(mg/dL)			non-survivor	12,54		

Ferritin	538	9-40000	survivor	192,0	345,5	<0,001
(ng/mL)			non-survivor	861,0		

D-Dimer	1130	0,52-6160	survivor	880,0	1095,0	0,252
(ng/mL)			non-survivor	1575,0		

Troponin-I	22,9	10-932	survivor	10,0	466,0	<0,001
(ng/mL)			non-survivor	44,8		

Procalcitonin	0,13	0,05-28,34	survivor	0,05	299,5	<0,001
(ng/mL)			non-survivor	0,62		

AKI; Acute kidney injury, CKD; Chronic kidney disease; Renal Tx; Renal transplantation.

**Fig. 1:**
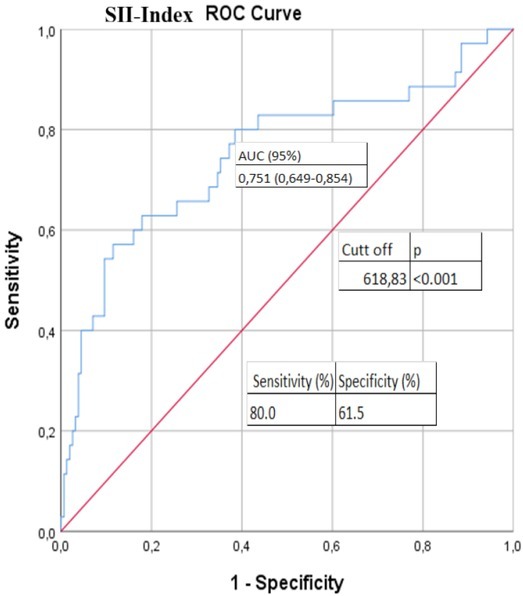
**ROC curve analysis to test diagnostic ability of SII index for mortality**. SII index: systemic immune-inflammation index, ROC: receiver operating characteristic curve; AUC: area under the curve.

There was a highly positive correlation between the SII and PNL, NLR, and PLR (r=0.754, p<0.001; r=0.812, p<0.001; r=0.841, p<0.001, respectively), while a moderate, positive correlation was found between the SII and white blood cell (WBC) count, RDW, and PLT (r=0.660, p<0.001; r=0.396, p<0.001; r=0.551, p<0.001, respectively). In addition, a moderate, negative correlation between the SII and lymphocyte count and a weak, negative correlation between the SII and hemoglobin was observed (r=-0.350, p<0.001; r=-0.199, p=0.006, respectively). No significant correlation was found between the SII and eGFR and creatinine levels, ferritin, procalcitonin, D-dimer, and troponin levels (p>0.05 for all). However, we observed a moderate, positive correlation between the SII and CRP values (r=0.439, p<0.001). Furthermore, there was a significant correlation between the SII and mortality (U=1,357, p<0.001), between NLR and mortality (U=1038.5, p<0.001), and between PLR and mortality (U=1144.0, p<0.001). The cut-off value of SII was 618.8 (area under the curve [AUC]=0.751, p<0.001) with 80.0% sensitivity and 61.5% specificity (Figure 1). A cut-off value of >618.8 was associated with a 4.68-fold higher mortality, increasing the mortality from 6.8% to 31.8% (Table 3).

**Table 3:** Complete blood count and hematological parameters

	Total median/ min-max	Survivor (median)	Non-survivor (median)		N	%	Mortality rate (%)	U	p
WBC	6000	6000	5700	<4500	41	21.5		2580,0	0,612
(x106/L)	(1500-19000)			4500-10500	135	70.7			
				>10500	15	7.8			

Lymphocyte	1200	1355	700	<1000	77	40,3		988,0	**<0,001**
(x106/L)	(200-3600)			>1001	114	59,7			

Neutrophil	3970	3835,0	4750,0					1942,5	**0,008**
(x106/L)	(290-16260)								

Eosinophil	10,0	20,0	10,0					2042,0	**0,018**
(x106/L)	(0,00-640,0)								
	Total (median)	Survivor (median)	Non-survivor (median)	Cut-off	Total N	%			

Hemoglobin	13,3	13,55	10,90					871,0	**<0,001**
(g/dL)	(5,7-17,4)								

PLT	178,0	178,0	178,0					2639,5	0,759
(x109/L)	(14-653)								

RDW	13,5	13,46	15,14					1458,0	**<0,001**
(%)	(2,6-24,0)	(±1,56)	(±2,43)						

SII-Index	585,96	495,52	1477,78	<618,8	103	53.9	6.8%	1357,0	**<0,001**
	(27,84-15062,5)			>618,9	88	46.1	31.8%		

NLR	3,31	2,73	9,27	<4,21	123	64.4	6.5%	1038,5	**<0,001**
	(0,29-43,8)			>4,21	68	35.6	39.7%		

PLR	151,46	139,94	287,5	<189,5	132	69.1	6.1%	1144,0	**<0,001**
	(16,09-1425,0)			>189,6	59	30.9	45.8%		

SD: standard deviation; WBC: white blood cell; RDW: red cell distribution width; PLT: platelets; NLR: neutrophil-to-lymphocyte ratio; PLR: platelet-to-lymphocyte ratio; SII-Index: Systemic immune-inflammation index.

**Figure 2:**
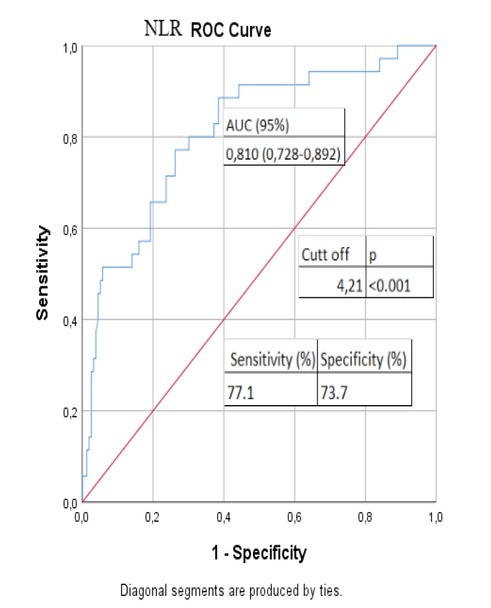
ROC curve analysis to test diagnostic ability of NLR for mortality NLR: neutrophil-to-lymphocyte ratio; ROC: receiver operating characteristic curve; AUC: area under the curve.

In addition, the cut-off value of NLR was calculated as 4.21 in this study. The significant correlation between the NLR and mortality considerably increased above this cut-off value (p<0.001) (Figure 2). Multivariate logistic regression analysis was performed for LDH, eGFR, CRP, ferritin, troponin-T, procalcitonin, age and SII, which were found to have a significant effect on mortality. We found that advanced age (OR:1.093; CI:%95 0.997-1.198; p=0.045), increase in procalcitonin level (OR:77.264; CI %95 1.926-3099.61; p=0.021) and increase in SII (OR:1.001; CI %95 1.000-1.004; p=0,040) are independent predictors of mortality.

## Discussion

COVID-19 is an infectious disease caused by a newly identified virus, namely SARS-CoV-2. It is a single-stranded ribonucleic acid (RNA) genome and belongs to genus *betacoronavirus* [11]. It enters the pulmonary epithelial cells by binding to angiotensin-converting enzyme 2 (ACE2) receptors and induces viral replication, leading to apoptosis of alveolar type 2 epithelial cells [12]. Clinical presentation may widely vary from asymptomatic or mild-to-moderate disease to severe disease, critical illness, and mortality [13]. As in all infectious diseases, early diagnosis is crucial to an effective management of the disease, early isolation, and early ICU decision. Therefore, several attempts have been made to identify biomarkers for early diagnosis, particularly for critically ill patients. A biomarker should be simple-to-use, give results rapidly, and become available in every center. In our study, we investigated whether the SII could predict mortality in patients with COVID-19. Our study results showed that SII can be used as an inflammatory marker to determine critical disease development or mortality in COVID-19 patients.

Since the aim of the study was to detect severe and critical patients beforehand, all patients were recruited from patients followed in the internal medicine service. SII was calculated using blood counts taken at the time of admission to the hospital. However, over time, patients who needed respiratory support were taken to the intensive care unit. Patients whose oxygen needs could not be met with nasal oxygen and/ or high flow were followed up with non-invasive or invasive mechanical ventilation when necessary. Vasopressor supplements were administered to patients with impaired hemodynamics.

The SII is a proinflammatory marker of systemic inflammation, such as NLR and PLR. Recently, hematological inflammatory markers have become a matter of interest for many researchers [14]. The SII and NLR have been already used as markers of systemic diseases and malignancy; however, recently, they have been increasingly utilized as the markers of systemic inflammation. Recently, studies have reported that SII can be used to determine activity in patients with Behçet‘s disease and ankylosing spondylitis [15, 16]. It has also been reported that SII can be used to predict the poor prognosis of antineutrophil cytoplasmic antibody-associated vasculitis [17]. Previous studies have extensively examined the lymphopenia, RDW, NLR, PLR, CRP, eosinopenia, and elevated lactate dehydrogenase (LDH) as predictors of mortality [18, 19, 20]. In the present study, there was a highly positive correlation between the SII and PNL, NLR, and PLR (r=0.754, p<0.001; r=0.812, p<0.001; r=0.841, p<0.001, respectively), while a moderate, positive correlation was found between the SII and CRP (r=0.439, p<0.001). These findings indicate that SII can be used as an acute phase reactant. In addition, we observed that the SII scores were significantly higher among the non-survivors than survivors, suggesting a significant relationship between the index scores and mortality. According to the ROC curve analysis, a cut-off value of SII was calculated as 618.8 (AUC=0.751, p<0.001) with 80.0% sensitivity and 61.5% specificity. A cut-off value of >618.8 was associated with a nearly 4.7-fold higher mortality. When multivariate logistic regression analysis was performed, we found that an increase in the SII (OR:1.001; CI 95% 1.000-1.004; p=0.040) was an independent predictor of mortality. Similarly, Xue et al. demonstrated in their study that there was a positive correlation between the derived neutrophil-to-lymphocyte ratio, the high-sensitivity C-reactive protein-albumin ratio and the SII with COVID-19 severity [21]. In the study of Fois et al., it was determined that SII can be used as a biomarker showing increased mortality [22]. Salman et al. demonstrated that increased levels of interleukin 6, CRP, and SII lead to more pronounced progress and increased intubation and mortality rates [23]. As a result of our study, we concluded that the SII obtained from the complete blood counts of COVID-19 patients at the time of admission to the hospital can predict disease progression, disease severity and mortality. In addition, the cut-off value of NLR was calculated as 4.21 in this study. The significant correlation between the NLR and mortality considerably increased above this cut-off value. These results are consistent with previous findings of Liu et al. [6]. In the study of Leonardo et al., a significant correlation was found between mortality with RDW and APACHE-II score [24]. In their study, Vogels et al. revealed that the SOFA score is an independent predictor of mortality in COVID-19 patients [25]. We also used the rapid Sequential Organ Failure Assessment (qSOFA) score, which is generally used to predict in-hospital mortality and intensive care unit admission in patients with sepsis, in our study [26]. The qSOFA score of non-survivors (mean:2.4±0.6) was significantly higher than the qSOFA score of surviving patients (mean:0.33±0.5) (t=-21,8; p<0.001). We also found a significant positive correlation between the qSOFA score and mortality (r=0,73; p<0.001). There was a significantly positive correlation between qSOFA score and SII (r=0.43; p<0.001). Similarly, we found a moderate positive correlation between RDW and SII-Index in our study (r=0,396, p<0,001). The RDW values of the patients who died were significantly higher, and we found a significant relationship between RDW and mortality (p<0,001). However, due to both the retrospective nature of our study and the lack of data to use to calculate the scores, we were unable to use SOFA and APACHE II scores.

In a meta-analysis, Zheng et al. [27], revealed that advanced age was significantly associated with the increased mortality. Similarly, the current study showed that advanced age significantly increased mortality and 82% of deceased patients were above 65 years of age.

In the present study, the mortality rate was comparable between male and female patients (18.1% *vs*. 18.6%, respectively). Unlike previous studies [28], we found no significant correlation between the sex and mortality (χ2=0.007, p=0.933). However, the most common symptoms were fever (70.7%), dry cough (61.3%), fatigue (56.5%), and dyspnea (33.5%) in our study, consistent with previous studies [29]. Similar to the study of Zhang et al. [30], we observed a significant correlation between the presence of dyspnea and the increased mortality (χ2=23.65, p<0.001).

Furthermore, the rate of patients (88%, n=168) having thoracic CT abnormalities at the time of admission was consistent with a previous study of Wu et al. [31]. Twenty-three (12.0%) patients having normal CT scans survived, while consolidation on thoracic CT was associated with the increased mortality (χ2=8.67, p<0.01).

Among hospitalized patients with COVID-19, AKI may develop secondary to hypovolemia, right heart failure, congestive heart failure, hemodynamic instability, drug-induced nephrotoxicity, nosocomial sepsis, increased angiotensin II, and direct cytopathic effects of SARS-CoV-2. In a study, Gabarre et al. [32] found the AKI incidence to be 25% in critically ill patients. In our study, this rate was 16.2% (n=31) with a 4.2-fold increase in mortality rates in these patients (χ2=22.35, p<0.001).

In the present study, the most common comorbidities were hypertension (37.7%), diabetes (23.0%), chronic renal failure (14.7%), and heart failure (7.9%) which all significantly increased the mortality rate. These findings are consistent with the results of Luo et al. [33].

Furthermore, elevated troponin I and LDH levels, which are specific cardiac markers, were found to significantly increase mortality, consistent with the study of Li et al. [34]. Similar to previous studies, we found a significant correlation between the ferritin and procalcitonin levels, acute inflammatory biomarkers, and the increased mortality [35]. Unlike previous studies showing a relationship between elevated D-dimer levels and the increased mortality [36], we found no significant correlation in our study.

The main limitations of this study are its retrospective design with a non-equal sample size between the study centers and between survivors and non-survivors.

## Conclusion

In conclusion, the present study showed a significant correlation between the SII and frequently used acute phase reactants such as PNL, NLR, PLR, CRP, and ferritin. Based on these findings, we suggest that the SII can be used as a proinflammatory marker of systemic inflammation, malignancy, infections, and rheumatic diseases for follow-up and prognosis. It can be effectively used independently of other markers to predict critical illness or mortality among COVID-19 patients. However, there is a need for studies involving larger-scale patient numbers in this regard.
